# Neural Plasticity in Obesity and Psychiatric Disorders 

**DOI:** 10.1155/2016/6053871

**Published:** 2016-04-07

**Authors:** Mauricio Arcos-Burgos, Maria T. Acosta, Ariel F. Martinez, Maximilian Muenke, Pablo J. Enriori, Claudio A. Mastronardi

**Affiliations:** ^1^Genomics and Predictive Medicine Group, Department of Genome Sciences, John Curtin School of Medical Research, The Australian National University, Canberra, ACT 2601, Australia; ^2^National Human Genome Research Institute, N.I.H, Bethesda, MD 20892, USA; ^3^Department of Neurology and Pediatrics, George Washington University, Washington, DC 20052, USA; ^4^Department of Physiology, Monash University, Melbourne, VIC 3800, Australia

The global burden of obesity and psychiatric disorders poses some of the greatest challenges to healthcare systems worldwide. These pathological conditions result from a combination of genetic and environmental factors. For instance, obesity and depression have a heritability of approximately 50% and are negatively affected by chronic stress, neuroendocrine and metabolic disorders, sedentary lifestyle, poor-quality diet, and excessive consumption of alcohol and other drugs of abuse [1, 2]. Interestingly, there is evidence suggesting a bidirectional negative interaction between these two pathological conditions [3, 4].

Recent evidence suggests that obesity and psychiatric disorders can reshape the brain. Whereas the hippocampus is a major brain area associated with mood and memory, the hypothalamus is the essential neuroendocrine region that controls food intake and energy expenditure. Previous research has shown that depressive behavior decreases adult hippocampal neurogenesis and antidepressant use prevents this effect [5]. Also, the adult hypothalamus can undergo neurogenesis and neurodegeneration [6]. There are common neuroinflammatory processes and cellular mechanisms occurring within different areas of the brain during obesity and psychiatric disorders [7]. Thus, the brain plasticity changes associated with obesity or psychiatric conditions could potentially expand the knowledge of both fields.

The review article entitled “Obesity Reduces Cognitive and Motor Functions across the Lifespan” by C. Wang et al. highlights the negative impact of obesity on cognition and motor control. The authors discuss the deleterious effects obesity-related brain plasticity and the central actions of leptin, BDNF, and IGF-1. Their review also addresses the impact of exercise on counteracting obesity and obesity-related deficits altering cognition and motor control. Thus, their contribution dwells into different possible interrelationships among obesity, diabetes, and central nervous system pathways and how environmental factors such as physical exercise could promote favorable outcomes.

In the review article entitled “The Effects of Leptin Replacement on Neural Plasticity,” G. Paz-Filho discusses the metabolic and neurocognitive effects of leptin replacement therapy. This field is currently gaining momentum since metreleptin, a leptin analogue, is currently being used to treat leptin-deficient individuals and also patients undergoing lipodystrophy and hypothalamic amenorrhea. This comprehensive review also describes leptin roles in neural plasticity and microglial function in leptin-deficient and leptin-sufficient individuals. The most relevant challenges to this treatment and its side effects are also described.

There is an ongoing bias in the scientific literature that frequently does not acknowledge sex differences in study designs and analyses. Since sex-dependent variance underlying obesity, metabolic syndromes, and neural plasticity is not an exception to this rule, E. Underwood and L. T. Thompson report novel data addressing some of these issues in rodents. In their manuscript entitled “A High-fat Diet Causes Impairment in Hippocampal Memory and Sex-Dependent Alterations in Peripheral Metabolism,” they show for the first time sex-dependent metabolic regulation occurring along with sex-independent cognitive behavioral performance.

Because current therapeutic strategies to treat obesity have failed in decreasing its global prevalence and incidence, novel approaches are much needed. In the review article entitled “Deep Brain Stimulation for Obesity: From a Theoretical Framework to Practical Application” R. K. Nangunoori et al. describe the central feeding and reward brain circuitries. The authors also postulate novel potential uses of deep brain stimulation to target the reward circuitry alone or in conjunction with hypothalamic areas modulating feeding behavior. However, further research in this area is needed to establish the putative use of this therapeutic approach.

Alzheimer's disease is a neurodegenerative disease that has been estimated to affect up to 35 million people worldwide and predicted to affect 1 in 85 individuals by 2050. In the paper entitled “A Mutation in DAOA Modifies the Age of Onset in PSEN1 E280A Alzheimer's Disease,” J. Velez et al. provide novel data identifying new genes that could regulate the age of onset of Alzheimer's disease. Their study is based on the largest pedigree segregating a unique form of Alzheimer's disease. The pedigrees have been ascertained from a genetic isolate in Colombia that the authors have been studying for over 30 years [8, 9]. This original paper complements the results of other recently published reports by the same team that describe a novel gene network regulating the age of onset of Alzheimer's disease in the same genetic isolate [10].

Neuropsychiatric conditions such as major depression have been associated with altered levels of central excitatory neurotransmitters and/or their receptors. L.-C. Lee et al. expanded the field of knowledge in this area by showing novel associations between major depression and disruptive behaviors and single-nucleotide polymorphisms (SNPs) located within the genes encoding NMDA receptors (NMDAr). Their results are reported in the paper entitled “Influence of Genetic Variants of the N-Methyl-D-Aspartate Receptor on Emotion and Social Behavior in Adolescents.” The authors recruited 832 adolescents to evaluate their emotional and social impairments and to investigate their possible association with 59 SNPs harbored in NMDAr genes.

The long-term effects of adverse early-life experiences have been frequently modelled in rodent paradigms of maternal separation. In the original article entitled “Repeated Three-Hour Maternal Separation Induces Depression-Like Behavior and Affects the Expression of Hippocampal Plasticity-Related Proteins in C57BL/6N Mice,” Y. Bian et al. report novel results using the maternal separation paradigm. The authors investigated the effect of maternal separation stress on hippocampal neuroplasticity during the early phase of young-adulthood development and found that mice had reduced levels of plasticity-related proteins and developed depressive-like behavior before reaching adulthood.

Finally, in the original article entitled “History of Illicit Stimulant Use Is Not Associated with Long-Lasting Changes in Learning of Fine Motor Skills in Humans,” G. Todd et al. studied the possible long-lasting effects of illicit stimulant use in putative changes in learning of fine motor skills. The authors assessed motor learning with a three-minute visuomotor tracking task in three groups of individuals: abstinent stimulant users and two control groups comprising non-drug users and cannabis users. Their results show that the ability of learning of a new fine visuomotor skill remains unchanged in individuals with a history of illicit stimulant use.

We hope that this special issue will provide new insights into important aspects of neural plasticity associated with obesity, stress, and drug abuse. We believe that the different research perspectives presented in this special issue will encourage efforts towards the development of novel scientific approaches to tackle obesity and psychiatric disorders such as major depression and addictive behavior. These chronic pathological conditions are responsible for one of the greatest global health and financial burdens of all times.
